# Preoperative Assessment of Patients Undergoing Bariatric Sleeve Gastrectomy: A Cross-Sectional Study

**DOI:** 10.1155/2022/3622119

**Published:** 2022-07-18

**Authors:** M. R. Tawfik, N. F. Aldawas, N. S. Almegbil, A. A. Bin Hamad, A. N. Alanazi, A. M. Alaidaroos, T. M. AlRawaf, A. A. Fayed

**Affiliations:** ^1^King Abdullah Bin Abdulaziz University Hospital, Riyadh, Saudi Arabia; ^2^Hepatobiliary Unit, Internal Medicine Department, Alexandria Faculty of Medicine, Alexandria University, Alexandria, Egypt; ^3^Clinical Sciences Department, College of Medicine, Princess Nourah Bint Abdulrahman University, Riyadh, Saudi Arabia

## Abstract

**Introduction:**

Saudi Arabia (SA) is one of the top countries in the world when it comes to the number of bariatric procedures performed each year. There is still some debate on whether to do regular or selective upper endoscopy during the preoperative examination. The purpose of this study was to explore various endoscopic findings and Helicobacter pylori (HP) infection in symptomatic and asymptomatic patients having laparoscopic sleeve gastrectomy (LSG) prior to surgery.

**Methods:**

We investigated a cohort of 132 patients referred to the endoscopy unit from the bariatric surgery outpatient clinic for prebariatric esophagogastroduodenoscopy (EGD) as a part of preoperative LSG. Data extraction from medical records included clinical data such as body mass index (BMI), gastrointestinal symptoms (that include heartburn, regurgitation, epigastric pain, and nausea), medical comorbidities, and laboratory investigations. It included data about the endoscopic findings of EGD procedure as esophageal, gastric, and duodenal findings results as well as the results of biopsy specimens that were taken.

**Results:**

Out of 132 patients, 29 (22%) had a BMI of less than 40 kg/m^2^ whereas 103 (78%) had a BMI of 40 kg/m^2^ or above, with an average of 44.4 ± 6.4 kg/m^2^. The average age of participants was 33.6 ± 10.4 years. HP was detected in 36 patients (35.0%) with a slightly greater prevalence in patients with a higher BMI (33.7%) than in patients with lower BMI (35.0%). Collectively, 73 patients (55.7%) had positive endoscopic findings of various grades, sites, and combinations. Incompetent cardia (35.6%) was the most often seen esophageal finding, antral gastritis (34.1%) was the most frequently encountered gastric finding, and duodenitis 1st part was the commonest duodenal endoscopic finding (7.8%). Among asymptomatic patients, incompetent cardia was detected in 33.3%, antral gastritis was found in 30.1%, and around one-quarter of them were positive on HP testing (26.6%). Additionally, 16.1% of them had signs of reflux esophagitis, 17.2% had hiatal hernia, and 14.0% had nodular gastritis.

**Conclusion:**

The current study revealed a high prevalence of positive endoscopic findings as well as HP infection upon routine endoscopic examination among patients undergoing bariatric surgery even those who were asymptomatic from any gastrointestinal symptoms.

## 1. Introduction

Saudi Arabia (SA) is experiencing a growing obesity crisis, which has been observed at a regional and national level, in different age groups, and among both males and females [1]. It has resulted from less regular exercise or physical activities, increased consumption of western fast foods, and community platters [2]. The study of the current trends and the future projection of adult obesity prevalence in Saudi Arabia showed that overall obesity will increase to 41% in men and 78% in women by the year 2022 [3]. This problem requires a focus on health measures [1].

Bariatric surgery is the only effective procedure that provides long-term sustained weight loss and improves obesity-related complications. However, there has been a rising incidence in complications within the acceptable range associated with these procedures including gastroesophageal reflux disease (GERD), nutrition, malabsorption, and dumping syndrome. Laparoscopic sleeve gastrectomy (LSG) has emerged over the last few years to be a common bariatric procedure with several advantages compared to more complex bariatric procedures, including avoiding an intestinal bypass with the resulting significant malabsorption [4, 5].

While people with morbid obesity or increased body mass index (BMI) are most likely to have GERD, the type of bariatric surgery should be selected according to their condition. The prevalence of clinically relevant GERD associated with bariatric surgery is variable but has been reported as ranging between 7% and 14%. LSG could make symptoms even more aggravated, while gastric bypass resolves symptoms of GERD, so the LSG is not recommended for known moderate to severe GERD and Barrett's esophagus (BE) patients. The prebariatric evaluation may change the surgical decision of the most suitable surgical method [6].

However, uncertainty remains regarding the exact prevalence of clinical situations like GERD or Helicobacter pylori (HP) infection in asymptomatic patients undergoing bariatric surgery as well as the case burden that may be due to differences in background characteristics of patient populations as well as the clinical phenotypes of the patient population studied; this study is aimed at exploring the prevalence of different endoscopic findings, the prevalence of HP in preoperative patients undergoing bariatric surgery, the associations between HP and endoscopic findings, and the correlations between endoscopic findings in symptomatic and asymptomatic patients in a sample of Saudi population.

## 2. Materials and Methods

### 2.1. Study Design

This was a cross-sectional study that was conducted on all patients referred to the endoscopy unit from the bariatric surgery outpatient clinic for prebariatric sleeve gastrectomy evaluation in the period from June 2019 to March 2020. The study was approved by the Institutional Review Board of Princess Nourah Bint Abdulrahman University (PNU) (approval number 19-0228). As the data was extracted from the medical records of patients anonymously, the written informed consent was waived in this study.

### 2.2. Study Population and Setting

This study included 132 patients, who underwent endoscopic esophagogastroduodenoscopy (EGD) as a part of preoperative LSG. It was conducted in Gastroenterology Unit, Internal Medicine Department, King Abdullah Bin Abdulaziz University Hospital (KAAUH), PNU-Riyadh, SA. Our hospital is a newly established university medical center that provides free bariatric surgery to all qualifying patients in addition to other medical specializations to encourage a healthy lifestyle with a special focus on obesity treatment.

Our protocol inclusion criteria for bariatric surgery include adults with BMI > 40 kg/m^2^, ≥35 kg/m^2^ with comorbidities (type 2 diabetes mellitus, hypertension, and obstructive sleep apnea), and adolescents with BMI ≥ 35 kg/m^2^ [7, 8]. All patients underwent preoperative assessment that includes medical, endocrinal, nutritional, and psychosocial evaluations as well as EGD assessment under conscious sedation (GIF-160, Olympus endoscopy, Tokyo, Japan). The eligible candidates were tested for HP infection by any of the available diagnostic methods (endoscopic biopsy, HP stool Ag, or urea breath test (UBT). Endoscopic biopsy specimens were taken from the gastric antrum and corpus in patients undergoing EGD, fixed in 10% formalin, and embedded in paraffin if they were taken.

Abstracting of data was done by using a data extraction sheet for all patients who had undergone prebariatric gastroscopy assessment from their medical records. All adult patients (above 18 years) were eligible to be included in the study with no gender restriction. Data extraction from medical records included clinical data such as weight, height, BMI, gastrointestinal symptoms (that include heartburn, regurgitation, epigastric pain, nausea, and the atypical symptoms of GERD), medical comorbidities, and the laboratory investigations. It included data about the endoscopic findings of EGD procedure as esophageal, gastric, and duodenal findings results as well as the results of biopsy specimens that taken.

### 2.3. Sampling Technique and Sample Size Calculation

We adopted a purposive nonprobability sampling technique. The sample size was calculated by STATA14 software based on the expected prevalence of positive endoscopic findings among asymptomatic patients ranging between 25 and 35%, a confidence level of 95% (alpha = 0.05), power of study of 80% (beta = 20%), and the minimal estimated sample size equal to 130.

### 2.4. Statistical Analysis

Descriptive statistics in terms of means, standard deviations, frequency, and percentages were used to describe the criteria of the studied sample. *T*-test (after confirming normality) was used to compare quantitative data while Chi-square/Fisher's exact test was used to compare qualitative data accordingly. SPSS (IBM version 26) software was used for analysis. A *P* value less than 0.05 was considered statistically significant.

## 3. Results

The study involved 132 patients, with 29 (22%) having a BMI of less than 40 kg/m^2^ with associated comorbidities and 103 (78%) having a BMI of 40 kg/m^2^ or above, with an average of 44.4 ± 6.4 kg/m^2^. The average age was 33.6 ± 10.4 years, and patients with a higher BMI were considerably younger (32.2 ± 9.6 compared to 38.9 ± 12.1, *P* value = 0.01) than those with a lower BMI. HP was detected in 36 patients (35.0%) with a slightly higher prevalence among patients with higher BMI (33.7%) than that reported among patients with smaller BMI (33.3%) (Table 1).

Collectively, 73 patients (55.7%) had positive endoscopic findings of various grads, sites, and combinations. Incompetent cardia (35.6%) was the most common esophageal endoscopic finding, followed by reflux esophagitis (18.6%), while antral gastritis (34.1%) was the most common gastric finding, and gastric ulceration (3.8%) was the least common gastric finding. Other gastric findings including bile reflux, antral erosions, and multiple gastric polyps were reported among 27 patients (20.6%). The most common duodenal endoscopic finding was duodenitis 1st part (7.8%), followed by duodenal ulceration (3.8%).

As compared to patients with BMI less than 40 kg/m^2^, patients with higher BMI reported a higher prevalence of hiatal hernia (19.6% versus 7.7%), antral gastritis (35.3% versus 30.8%), and other gastric findings (21.4% versus 17.9%) (Table 1).

Table 2 shows the different endoscopic and clinical findings among the studied sample according to the positivity of H. pylori. Among patients with positive H. pylori, the commonest esophageal prevalent finding was incompetent cardia (33.3%) while the least was hiatal hernia (11.1%), although both of them did not correlate with H. pylori.

Positive HP cases were significantly more prevalent among those with nodular gastritis (68.8%) and those with gastric ulceration (80.0%). Additionally, on biopsy examination, 91.7% of patients with positive HP had active chronic gastritis compared to only 22.5% of those with negative H. pylori. Only one patient had dysplasia on biopsy examination (Table 2).

Table 3 shows the endoscopic findings among the study participants according to their GI symptoms. About 70% of participants did not report symptoms, while only 28.8% suffered from GI symptoms. As expected, symptomatic patients had more positive endoscopic and biopsy findings, especially in esophageal and gastric areas. However, asymptomatic patients showed all positive endoscopic and biopsy findings despite not suffering from GI symptoms. About one-third of asymptomatic patients had incompetent cardia (33.3%), antral gastritis (30.1%), and active chronic gastritis (35.1%). Around one-quarter of them were positive on HP testing (26.6%) and even the patient who had dysplasia was asymptomatic. Additionally, 16.1% of them had signs of reflux esophagitis, 17.2% had hiatal hernia, and 14.0% had nodular gastritis.

## 4. Discussion

The current study revealed a high prevalence of positive endoscopic findings upon routine endoscopic examination among patients undergoing bariatric surgery even those who were asymptomatic from any GERD symptoms. The commonest endoscopic lesions were gastritis and incompetent cardia. There was also a high prevalence of HP infection among symptomatic and asymptomatic patients as well as among patients with and without gastric and esophageal lesions.

Bariatric surgery has become a common treatment for morbid obesity and its comorbidities. In the Global Registry Report 2018 of the international federation for the Surgery of Obesity (IFSO), Saudi Arabia, had a total case number of 3120 in the calendar year from 2013 to 2018, ranking at the 13th highest prevalence of bariatric surgery worldwide including the highest rates of laparoscopic sleeve gastrectomy procedures [9]. The question remains, whether the EGD for bariatric surgery candidates is a necessity to be performed before the surgical procedure if the patients were found to be asymptomatic upon clinical examination, as some surgeons feel it can delay the surgical procedure and increase the expense and they advocate selective endoscopy in symptomatic patients only [10, 11]. On the other hand, many surgeons consider endoscopic evaluation of the upper gastrointestinal tract before surgical alteration with a bariatric procedure is a requirement [12–14].

About 55% of the participants in the current study had upper GI lesions upon endoscopic examination, and a considerable proportion of those who were completely asymptomatic before examination had some positive endoscopic findings with various severity and histopathological gradings. These findings are in line with other research: Suter et al. found that a third of morbidly obese patients had symptoms of GERD, of which 53% had hiatal hernia and 31.4% had peptic esophagitis [15]. Some researchers reported a higher prevalence of positive endoscopic results among the bariatric patients reaching up to 81% in a recent Saudi study [16]. However, a meta-analysis published in 2016 concluded that 92% of the bariatric patients who underwent endoscopic evaluation had normal or insignificant lesions that led to complications or changes in the surgery plan [17].

Endoscopic findings vary in type/degree and histopathological grade across all available literature. Among our patients, incompetent cardia, antral gastritis, reflux esophagitis, and hiatal hernia were the commonest lesions. A large study done on 1369 bariatric patients showed that 40.1% of patients showed mild lesions (defined as mild esophagitis, gastritis, and/or duodenitis, esophageal web). They also reported other lesions at a lower extent as mucosal/submucosal mass lesions, ulcers, severe erosive esophagitis, gastritis, and/or duodenitis, bezoar, hiatal hernia, peptic stricture, Zenker's or esophageal diverticula, and arteriovenous malformations while the commonest findings was hiatal hernia or gastric polyp/s [11]. Another study reported that inactive chronic gastritis was the most common histopathologic finding, and intestinal metaplasia was identified in 8 patients (1.7%) [18]. Wilson et al. also showed that there was an association between obesity and hiatal hernia and reflux esophagitis [19]. Recently, a study from Saudi Arabia reported gastritis as the commonest finding [16]. The reason behind this scene might be due to the variations in the studied population characteristics (e.g., their BMI) as well as the adopted protocols in different centers as some centers adopt routine endoscopies for all bariatric patients and others follow selective protocol.

Nevertheless, it has been well demonstrated that the incidence of GERD symptoms in obese subjects does not correlate well with the severity of the disease [20, 21]. For this reason, some authors suggest the need to assess the presence of at least a hiatal hernia in the preoperative work-up for morbidly obese patients who are candidates for bariatric surgery as it can be corrected in the same operation [22, 23].

At the moment, the decision for a personalized preoperative endoscopy before the bariatric surgery is mostly owing to the increase in gastrointestinal diseases in obese people, which may affect the perioperative therapy or even the surgery itself [24]. In the current study, we found the prevalence of all endoscopic findings comparable between participants with high or very high BMI (more than or less than 40 kg/m^2^). Therefore, customizing the preoperative endoscopy for very obese patients deems quietly unwarranted.

HP prevalence in the countries located in the southwest and western Asia as well as North Africa is still high in the healthy asymptomatic population. There are few systematic reviews on the prevalence and epidemiology of HP in this geographically important region of the world, especially among patients who underwent LSG. We detected HP in 35% of our patients, with a little greater frequency in individuals with a higher BMI (33.7%) than that reported among other patients. Our rates are thought to be lower than those seen in previous studies. Turan and Kocaöz observed that 50.0% of the patients who had EGD were positive for HP on assessment of biopsy data in a large study of 1257 LSG patients [25]. Another study estimated the prevalence to be 46.67% [6]. Additionally, studies done in Saudi had the prevalence of HP at 41% and 85%, respectively, among their subjects [26, 27]. This gap might be attributed to differences in confirming HP positivity between studies or to demographic disparities.

Regarding HP, the main reasoning for screening in prebariatric surgery patients is to limit the occurrence of postoperative compilations such as marginal ulceration [28] and viscus perforation [29] as well as the reported difficult eradication after LSG. Taking that into consideration, the high estimates of HP prevalence in Saudi Arabia solidify the significant impact of EGD and HP screening on prebariatric patients.

In the light of, the higher percentage of a large range of upper GI tract disorders in obese patients in our study that were confirmed with similar studies done on histopathological specimens of LSG [30–32]. We recommend performing a routine preoperative endoscopic evaluation for patients before bariatric surgery even if they are asymptomatic, which may help in the diagnosis of GI pathologies affecting both the surgery and the bariatric follow-up.

### 4.1. Limitations

A limitation of this study would be the small sample size; however, complete endoscopic and laboratory findings for this cohort in a newly established center can be used for further comparisons and evaluation of patients' risk profiles and outcomes.

## Figures and Tables

**Table 1:** Clinical and endoscopic characteristics of the studied sample.

	All participants	BMI < 40 kg/m^2^ (*N* = 29)	BMI ≥ 40 kg/m^2^ (*N* = 103)	*P* value
Age	33.6 ± 10.4	38.9 ± 12.1	32.2 ± 9.6	0.01
Sex (males)	49 (37.1)	10 (34.5)	39 (37.9)	0.64
*Laboratory findings*				
HbA1c %	5.7 ± 1.3	5.6 ± 1.2	5.6 ± 1.2	0.90
TSH (*μ*L/mL)	2.5 ± 1.8	1.9 ± 1.1	2.6 ± 1.9	0.03^∗^
T4 (pmol/L)	13.8 ± 2.1	13.4 ± 1.7	13.8 ± 2.2	0.37
T3(pmol/L)	4.9 ± 0.8	4.5 ± 0.5	4.9 ± 0.9	0.02^∗^
LDL (mmol/L)	3.3 ± 0.7	3.3 ± 0.8	3.3 ± 0.8	0.76
HDL (mmol/L)	1.1 ± 0.2	1.2 ± 0.3	1.1 ± 0.2	0.14
TRI (mmol/L)	1.3 ± 0.7	1.5 ± 0.9	1.3 ± 0.6	0.33
Total cholesterol (mmol/L)	4.9 ± 0.8	4.9 ± 0.8	4.9 ± 0.8	0.95
Helicobacter pylori prevalence (%)	36 (35.0)	8 (33.3)	28 (33.7)	0.90
*Esophageal endoscopic findings* (%)				
Reflux esophagitis	24 (18.6)	5 (19.2)	19 (18.6)	0.87
Hiatal hernia	22 (17.1)	2 (7.7)	20 (19.6)	0.24
Incompetent cardia	46 (35.6)	11 (42.3)	35 (34.3)	0.45
*Gastric endoscopic findings* (%)				
Pangastritis	7 (5.4)	2 (7.7)	5 (4.9)	0.63
Antral gastritis	44 (34.1)	8 (30.8)	36 (35.3)	0.82
Nodular gastritis	16 (12.4)	4 (15.4)	12 (11.8)	0.62
Gastric ulceration	5 (3.8)	3 (11.5)	3 (2.9)	0.09
Other gastric findings	27 (20.7)	5 (17.9)	22 (21.4)	0.79
*Duodenal endoscopic findings* (%)				
Duodenitis 1^st^ part	10 (7.8)	1 (3.8)	9 (8.8)	0.68
Duodenitis 2^nd^ part	2 (1.6)	0 (0.0)	2 (2.0)	0.99
Duodenitis both parts	3 (2.3)	1 (3.8)	2 (2.0)	0.50
Duodenal ulcer	5 (3.8)	1 (3.8)	4 (3.9)	0.99

^∗^Significant *P*-value.

**Table 2:** Endoscopic findings among participants with and without HP infection.

	HP negative *N* = 71	HP positive *N* = 36	*P* value
*Esophageal findings*			
Reflux esophagitis	11 (15.5)	8 (22.2)	0.39
Hiatal hernia	11 (15.5)	4 (11.1)	0.53
Incompetent cardia	26 (36.6)	12 (33.3)	0.83^#^
*Gastric findings*			
Pangastritis	2 (2.8)	4 (11.1)	0.17^#^
Antral gastritis	25 (35.2)	15 (41.7)	0.52
Nodular gastritis	5 (7.0)	11 (30.6)	0.002^∗^
Gastric ulceration	1 (1.4)	4 (11.1)	0.04^∗^
Other gastric findings	13 (18.3)	9 (25.)	0.41
*Duodenal findings*			
Duodenitis 1^st^ part	4 (5.6)	6 (16.7)	0.09
Duodenitis 2^nd^ part	1 (1.4)	1 (2.8)	0.99
Duodenitis both parts	1 (1.4)	2 (5.6)	0.26
Duodenal ulcer	3 (4.2)	34 (5.6)	0.99
*Biopsy findings*			
Active chronic gastritis	16 (22.5)	33 (91.7)	<0.01
Dysplasia	1 (1.4)	0 (0.0)	0.70

^∗^Significant *P* value. ^#^Fisher exact test used.

**Table 3:** Endoscopic findings among symptomatic and asymptomatic participants.

	Asymptomatic (*N* = 94, 71.2%)	Symptomatic (*N* = 38, 28.8%)	*P* value
*Esophageal findings*			
Reflux esophagitis	15 (16.1)	9 (23.7)	0.22^#^
Hiatal hernia	16 (17.2)	7 (18.4)	0.86
Incompetent cardia	31 (33.3)	18 (47.4)	0.13
*Gastric findings*			
Pangastritis	6 (6.5)	1 (2.6)	0.67^#^
Antral gastritis	28 (30.1)	17 (44.7)	0.11
Nodular gastritis	13 (14.0)	3 (7.9)	0.33^#^
Gastric ulceration	5 (5.4)	1 (2.6)	0.67^#^
Other gastric findings	15 (16.1)	12 (31.6)	0.05
*Duodenal findings*			
Duodenitis 1^st^ part	7 (7.5)	4 (10.5)	0.57^#^
Duodenitis 2^nd^ part	2 (2.2)	0 (0.0)	0.36^#^
Duodenitis both parts	3 (3.2)	0 (0.0)	0.55^#^
Duodenal ulcer	3 (3.2)	2 (5.3)	0.62^#^
*Biopsy findings*			
Active chronic gastritis	33 (35.1)	16 (42.1)	0.67
Dysplasia	1 (1.1)	0 (0.0)	0.75
*HP infection*	25 (26.6)	11 (28.9)	0.54

Other gastric finding includes bile reflux, antral erosion, multiple gastric polyps. ^#^Fisher's exact test.

## Data Availability

The data supporting the findings and conclusions of the study are available upon request from the corresponding author.
